# Synuclein gamma expression enhances radiation resistance of breast cancer cells

**DOI:** 10.18632/oncotarget.25415

**Published:** 2018-06-08

**Authors:** Lu Tian, Yucui Zhao, Marie-José Truong, Chann Lagadec, Roland P. Bourette

**Affiliations:** ^1^ University of Lille, CNRS, Institut Pasteur de Lille, UMR 8161-M3T-Mechanisms of Tumorigenesis and Targeted Therapies, SIRIC ONCOLille, F-59000 Lille, France; ^2^ University of Lille, Inserm U908 Cell Plasticity & Cancer, F-59655 Villeneuve d’Ascq, France

**Keywords:** synuclein gamma, breast cancer, radiation, resistance, biomarker

## Abstract

Resistance to therapy is a major obstacle for the effective treatment of cancer. Expression of synuclein-gamma (SNCG) has been associated with poor prognosis and resistance to therapy. While reports on SNCG overexpression contributing to chemoresistance exist, limited information is available on the relationship between SNCG and radioresistance of cancer cells. Here we investigated the role of SNCG in radiation resistance in breast cancer cells. siRNA mediated knockdown of SNCG (siSNCG) markedly reduced SNCG protein level compared to scrambled siRNA (siScr) treatment. Furthermore, siSNCG treatment sensitized Estrogen Receptor-positive breast cancer cells (MCF7 and T47D) to ionizing radiation at 4 to 12 Gy as evidenced by the significant increase of apoptotic or senescent cells and reduction in clonogenic cell survival in siSNCG treated cells compared to siScr treated cells. On the other hand, we established an *in vitro* model of SNCG ectopic expression by using a triple-negative breast cancer cell line (SUM159PT) to further investigate the radioprotective effect of SNCG. We showed that ectopic expression of SNCG significantly decreased apoptosis of SUM159PT cells and enhanced clonogenic cell survival after radiation treatment. At the molecular level, after irradiation, the p53 pathway was less activated when SNCG was present. Conversely, p21^Waf1/Cip1^ expression was upregulated in SNCG-expressing cells. When p21 was down-regulated by siRNA, radiosensitivity of SNCG-expressing SUM159PT cells was dramatically increased. This suggested a possible connection between p21 and SNCG in radioresistance in these cells. In conclusion, our data provide for the first time experimental evidence for the role of SNCG in the radioresistance of breast cancer cells.

## INTRODUCTION

Breast cancer is the most frequently diagnosed cancer and one of the leading causes of cancer death in women worldwide. In 2012, the number of newly diagnosed cases in the world was estimated at 1.7 million, which represents 25% of all cancer cases in women [1]. Although dramatic advances have been made in the effectiveness of anti-cancer therapies, the death rate remains relatively high for breast cancer patients due to hard-to-treat metastatic and recurrent tumors. Radiation therapy is a useful cancer treatment strategy and is a highly cost-effective single-modality treatment. For *in situ* and infiltrating breast cancer, radiotherapy significantly reduces the risk of local recurrence and increases overall survival [2]. However, some patients do not show any benefit from this treatment due to individual variation in radiosensitivity. It is therefore necessary to develop new biomarkers that predict the effectiveness of radiotherapy.

Synuclein-γ (SNCG) is a member of the synuclein family which is a small, soluble, highly conserved group of neuronal proteins that have been implicated in both neurodegenerative diseases and cancer [3, 4]. It was first named breast cancer-specific gene 1 (BCSG1) due to its highly specific expression in advanced stages of breast cancer compared to its undetectable level in normal or benign breast lesions [5, 6]. Furthermore, abundant expression of SNCG has also been associated with several other types of cancer, including ovary, cervical, prostate, pancreatic, colon and lung [7–9]. In breast cancer, a series of functional studies have demonstrated that ectopic expression of SNCG in breast cancer cell lines promotes their proliferation as well as their ability to migrate and to metastasize [5, 10, 11]. At the same time, invalidation of SNCG in breast cancer cells sensitizes them to endoplasmic reticulum stress-induced apoptosis [12]. Moreover, the poor overall SNCG-related prognosis in breast cancer has also been reported [13, 14].

Previous studies have shown that the expression of SNCG confers resistance to anti-microtubule drugs used in breast cancer treatment, such as nocodazole or taxol [15, 16]. The reduced efficacy of these microtubules inhibitors is attributed to the SNCG-BubR1 interaction [11, 15]. SNCG has been shown to interact with BubR1, a mitotic checkpoint kinase required for the prevention of cell mitotic divisions following severe cell damage or mutation [11]. The SNCG-BubR1 interaction can prevent the activation of SAC (spindle assembly checkpoint) caused by microtubules inhibitors, and as a result, allowing cancer cells to progress into the cell cycle and escape apoptosis. Nevertheless, the relationship between SNCG expression and radiotherapeutic efficacy remains to be elucidated. A recent study of breast cancer patients with indications for postoperative radiotherapy suggested that high SNCG expression is an indication of fewer radiotherapeutic benefits [17]. However, the role of SNCG in radiotherapy resistance and its mechanism still need to be validated.

Here we show the potential use of SNCG as a biomarker to predict the effectiveness of radiotherapy in breast cancer patients. We used various breast cancer cell lines that are either SNCG-positive or SNCG-negative as an *in vitro* working model to study the correlation between SNCG expression and responses of cancer cells to radiation. We demonstrated the inverse relationship between SNCG expression and sensitivity to radiation of breast cancer cells.

## RESULTS

### Expression of SNCG in human breast cancer cell lines

**Figure 1:**
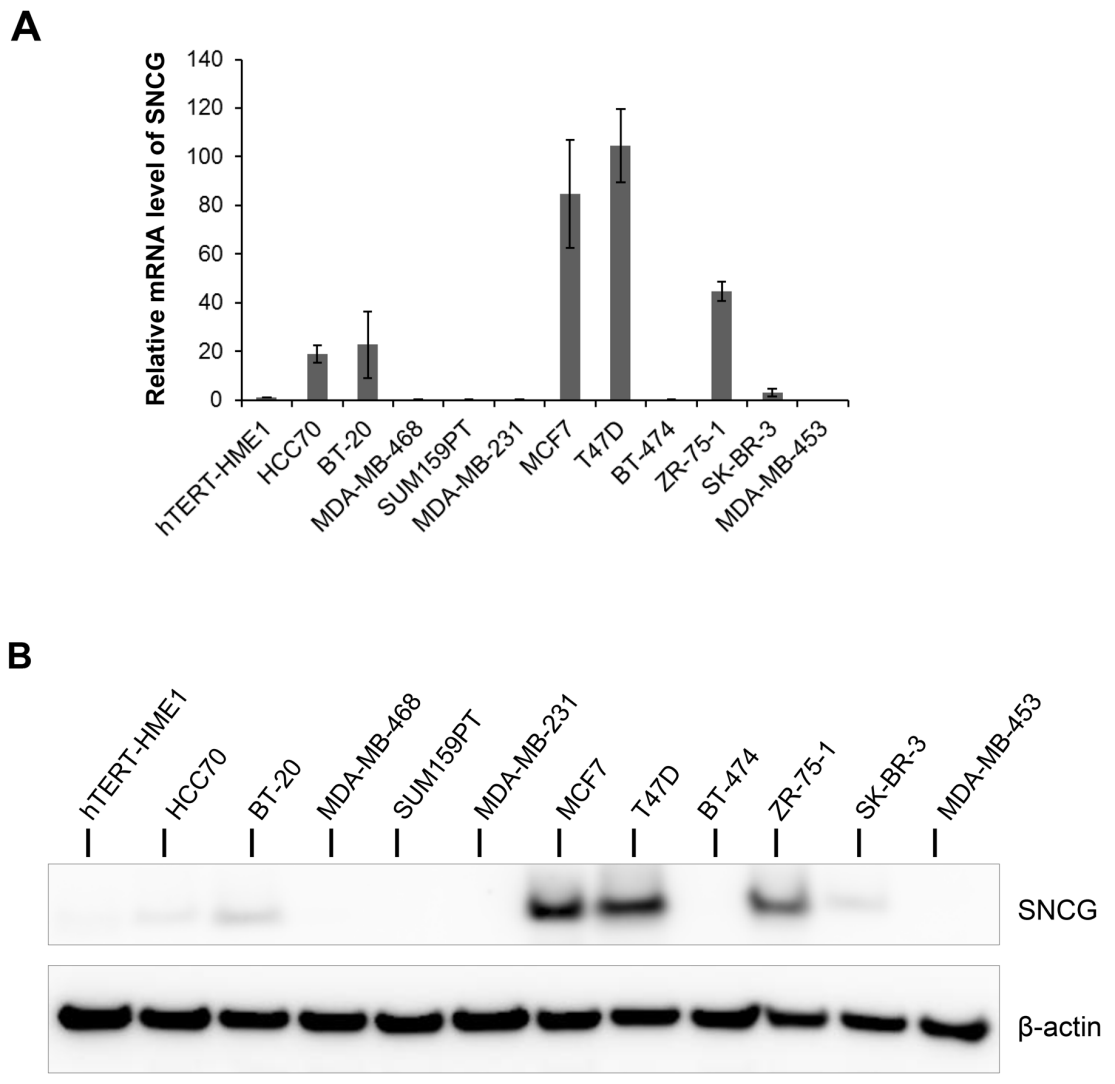
Expression of SNCG in breast cancer cell lines **(A)** Expression was analyzed by qRT-PCR using total RNA from different breast cancer cell lines. Levels of SNCG expression were normalized to those of RPLP0 internal control. Graph shows fold enrichment over normal immortalized breast epithelial hTERT-HME1 cells. Data represent the mean (± standard deviation, SD) of three independent experiments. **(B)** Representative immunoblot analysis (n=3) was performed on whole cell lysate for SNCG expression using anti β-actin antibody as a loading control.

Previous reports have suggested that SNCG is abnormally expressed in breast tumors and cell lines derived from breast tumors. We profiled a panel of *in vitro* breast cancer cell lines as well as hTERT-HME1 human mammary epithelial cells for SNCG expression both at transcript and protein levels. These cell lines belong to distinct breast cancer subtypes: luminal (MCF7, T47D, BT-474, ZR-75-1, SK-BR-3, MDA-MB-453), basal A (HCC70, BT-20, MDA-MB-468), and basal B (SUM159PT, MDA-MB-231) [18, 19]. As shown in Figure 1A, six cell lines expressed SNCG transcripts with the highest expression in T47D, MCF7, and ZR-75-1 luminal cells. Accordingly, when SNCG protein expression was investigated by immunoblotting, the 15 kDa endogenous SNCG protein was observed in T47D and MCF7 cells, and to a lesser extent in ZR-75-1 cells. Faint SNCG protein expression was observed in SK-BR-3, BT-20 and HCC70 cells (Figure 1B). On the contrary, SNCG protein was not detected in triple negative breast cancer cell lines, such as MDA-MB-468, MDA-MB-231, and SUM159PT cells, neither in hTERT-HME1 cells (Figure 1B).

### Down-regulation of SNCG increases cancer cell radiosensitivity

**Figure 2:**
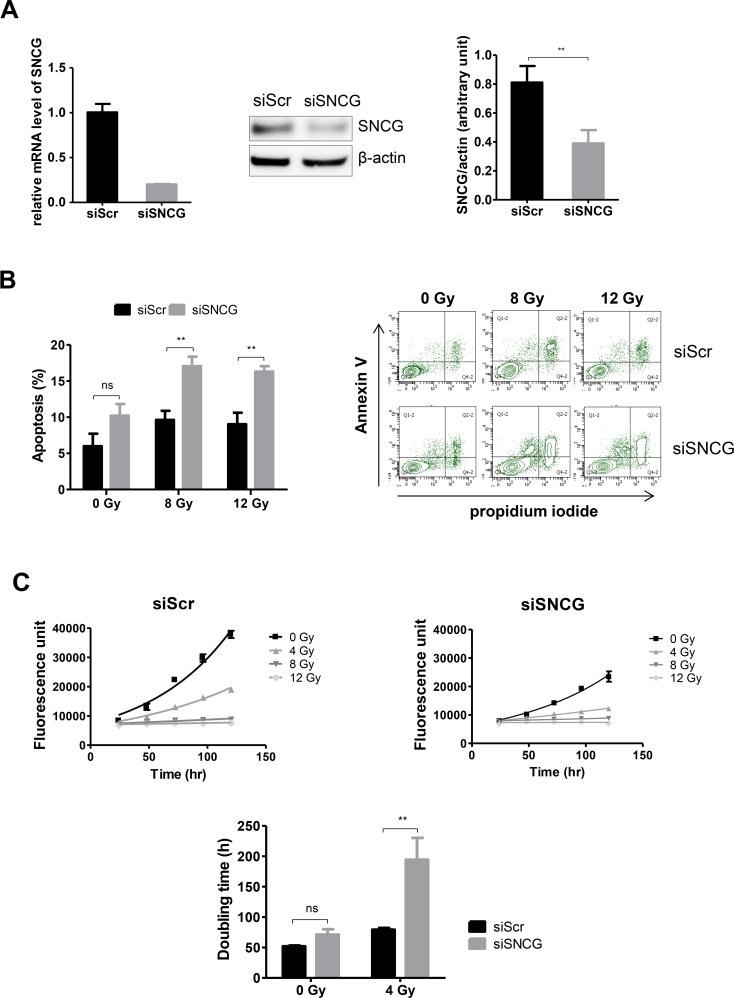
Inhibition of SNCG expression increases T47D cell radiosensitivity **(A)** siSNCG-treated T47D cells showed reduced RNA (left panel) and protein (middle and right panels) expression compared to siScramble (siScr)-treated T47D cells. Relative expression of SNCG mRNA (left panel) was assessed by qRT-PCR analysis performed in triplicate (normalized against RPLP0). Representative immunoblot analysis (middle panel) was performed on whole cell lysate for SNCG expression using anti β-actin antibody as a loading control. Bar graph (right panel) shows quantitative analysis of scanning densitometric values of SNCG protein as ratio to β-actin protein. Data represent mean values ± standard error of the mean of two (RNA) and three (protein) independent experiments. **(B)** siSNCG-treated T47D cells showed increased apoptosis after radiation treatment (8 and 12 Gy) compared to siScramble (siScr)-treated T47D cells. Flow cytometry analysis was carried out to detect apoptotic and necrotic cells. Histogram shows percentage of apoptotic cells 72 hours after irradiation (left panel). Data represent mean values ± standard error of the mean of four independent experiments. Representative experiment of flow cytometry analysis (n=4) shows the percentage of Annexin-V and propidium iodide staining of T47D cells irradiated or not at a dose of 8 or 12 Gy (right panel). **(C)** Radiation sensitivity was determined from the number of viable cells at different times after irradiation at 4, 8, and 12 Gy using the resazurin-based cell viability assay. The upper panel shows representative growth curves of siScr- or siSNCG-treated cells. Curves from three independent experiments were used as basis for calculation of doubling time in hours (hr) (lower panel) ^**^ = *P value* < 0.01; ns = not significant.

Previous experiments have demonstrated that inhibition of SNCG expression in T47D cells reduced their resistance to drugs [15, 20]. To determine whether the inhibition of SNCG expression could also decrease radioresistance of breast cancer cells, we delivered siRNA to knock down SNCG expression in T47D cells. As shown in Figure 2A, 48h after transfection both SNCG RNA (left panel), and protein levels (middle and right panels) were repressed till 20.2%±2% and 46.7%±10.4% of the negative control, siScramble (Scr)-treated cells, respectively. When SNCG siRNA-treated T47D cells were irradiated, a significant increase of apoptosis was obtained at 8 and 12 Gy as compared to irradiated siScr-treated cells (Figure 2B). Previous studies have shown that inhibition of endogenous SNCG expression dramatically diminished the clonogenicity of T47D cells [21, 22]. To further characterize radiosensitivity of siRNA-treated cells, we thus performed cell proliferation analysis using a colorimetric assay [23]. The proliferation of siSNCG-treated cells (doubling time of 71.2±8.8 hours) was not significantly different from the proliferation of control siScr-treated cells (doubling time of 52±2.3 hours) (Figure 2C). On the contrary, when cells were irradiated at 4 Gy, the proliferation of siSNCG-treated cells was significantly reduced (doubling time of 194.3±36 hours) as compared to siScr-treated cells (doubling time of 79.2±5.2 hours). At 8 and 12 Gy, no measurable proliferation was observed (Figure 2C, upper panels).

**Figure 3:**
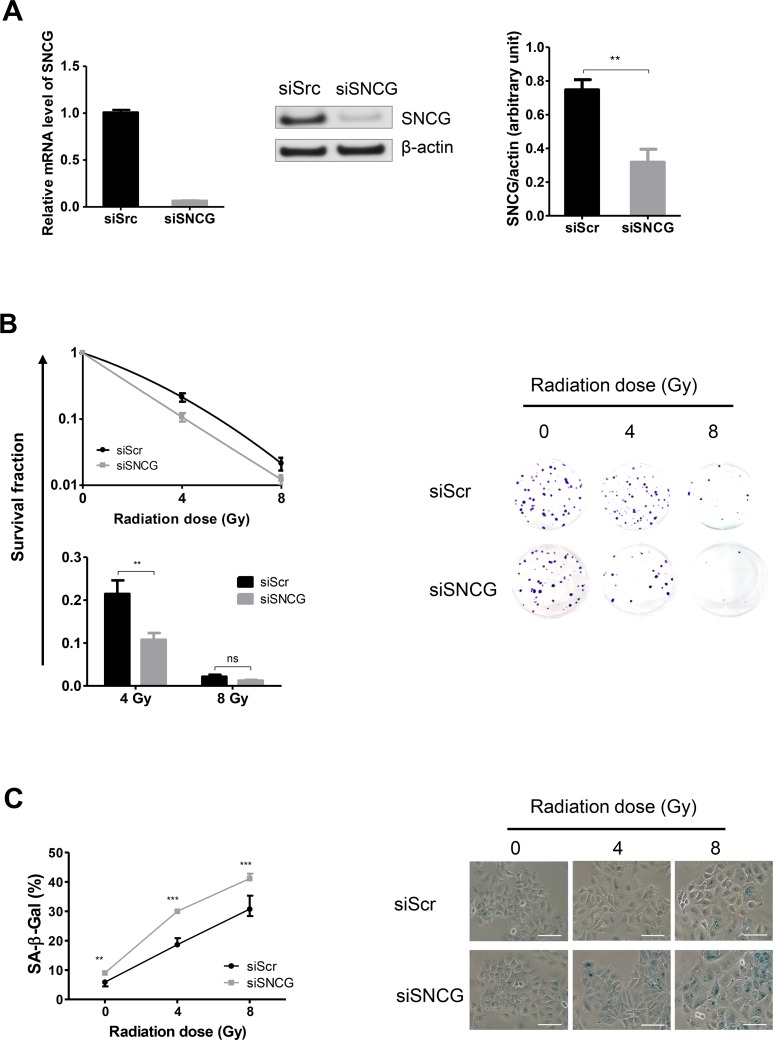
Inhibition of SNCG expression increases MCF7 cell radiosensitivity **(A)** siSNCG-treated MCF7 cells showed reduced RNA (left panel) and protein (middle and right panels) expression compared to siScramble (siScr)-treated cells. Relative expression of SNCG mRNA (left panel) was assessed by qRT-PCR analysis performed in triplicate (normalized against RPLP0). Representative immunoblot analysis (middle panel) was performed on whole cell lysate for SNCG expression using anti β-actin antibody as a loading control. Bar graph (right panel) shows quantitative analysis of scanning densitometric values of SNCG protein as ratio to β-actin protein. Data represent mean values ± standard error of the mean of two (RNA) and three (protein) independent experiments. **(B)** siSNCG-treated MCF7 cells showed decreased clonogenic potential after radiation treatments (4 and 8 Gy) compared to siScramble (siScr)-treated cells. Clonogenic cell survival assay was performed and curves show the percentage of survival after irradiation (left panel). Data represent mean values ± standard error of the mean of three independent experiments, each done in triplicate. Representative pictures (n=3) of 2-week-old colonies after fixation and crystal violet staining (right panel). **(C)** siSNCG-treated MCF7 cells showed increased cellular senescence after radiation treatments (4 and 8 Gy) compared to siScramble (siScr)-treated cells. Cellular senescence was evaluated by the detection of SA-β-galactosidase activity and curves show the percentage of SA-β-gal positive cells as mean values ± standard error of the mean of three independent experiments (left panel). Representative images of SA-β-gal positive cells (blue) are shown (right panel) (scale bar = 100 μm). ^***^ = *P value* < 0.005 ; ^**^ = *P value* < 0.01 ; ns = not significant.

We next investigated the outcome of SNCG silencing on the radiosensitivity of MCF7 cells by assessing their clonogenic survival potential. Similar to T47D cells, siSNCG-treated MCF7 cells exhibited a decrease in SNCG expression at both RNA (6.6%±0.7%) and protein (41.5%±12.3%) levels (Figure 3A). The clonogenic assay revealed that with increasing radiation doses (4 and 8 Gy), cell colony formation was reduced. In addition, survival fraction at 4 Gy was significantly reduced in siSNCG-treated MCF7 cells compared to siScr-treated MCF7 cells (Figure 3B, lower left panel). No significant difference in apoptosis was observed between siSNCG-treated and siScr-treated MCF7 cells (data not shown) and cell cycle analysis did not show any significant difference either (Figure Supplementary Figure 1A). It has been previously shown that an important pathway of radiation-induced cell death for MCF7 cells was replicative senescence [24, 25]. Cellular senescence in the presence of altered SNCG expression was then evaluated by measuring SA-β-galactosidase positive cells after irradiation. In the absence of irradiation, few SA-β-gal positive cells were observed in both siScr-treated and siSNCG-treated cell populations (Figure 3C). After irradiation, numbers of SA-β-gal positive cells were markedly increased in both cell populations. Notably, siSNCG-treated cells showed a higher number of SA-β-gal positive cells compared to siScr-treated MCF7 cells (Figure 3C). These results confirmed that cellular senescence is well induced in irradiated MCF7 cells and demonstrated that SNCG down-regulation amplified the senescence pathway. Altogether, these results demonstrated that inhibition of SNCG expression increased the sensitivity to radiation of both T47D and MCF7 cells.

### Expression of SNCG enhances cancer cell radioresistance

**Figure 4:**
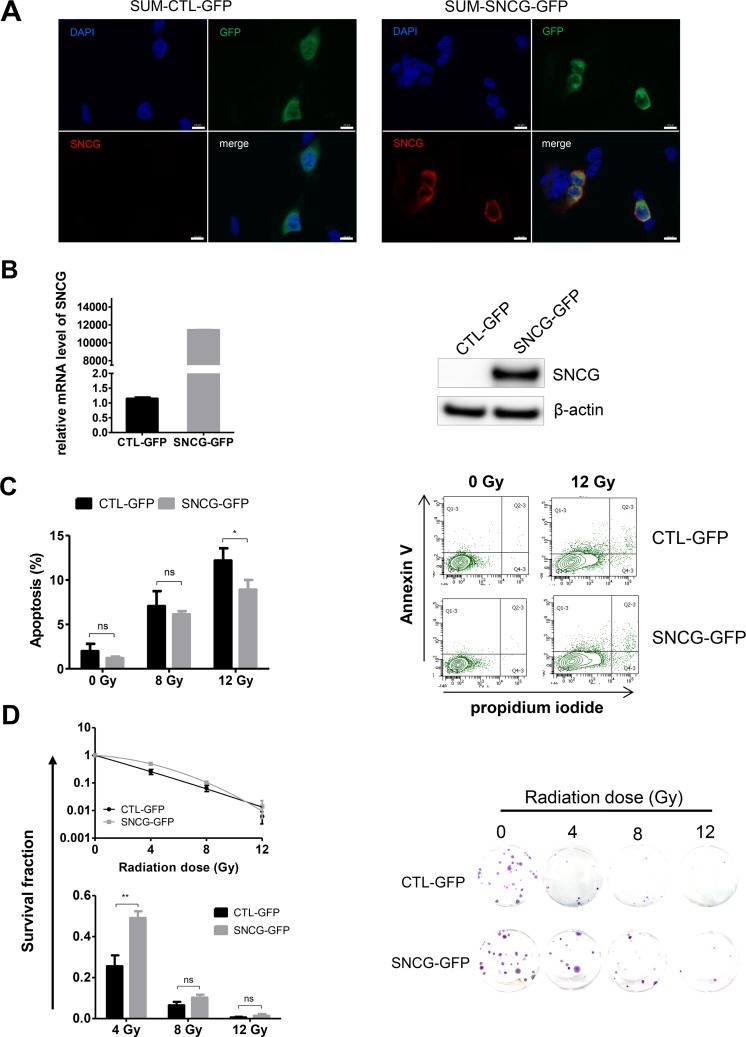
Ectopic expression of SNCG in SUM159PT cells increases their resistance to ionizing radiation **(A)** SUM159PT cells were transfected either with pCMV6-SNCG-GFP or pCMV6-A-GFP vector as a control (CTL) and GFP-positive cells were sorted by FACS. Representative photographs of SUM-CTL-GFP (left panels) and SUM-SNCG-GFP (right panels) cells expressing GFP (green) and stained with DAPI nuclear stain (blue); scale bar = 10 μm. **(B)** SNCG expression was analyzed by qRT-PCR using total RNA from SUM-CTL-GFP and SUM-SNCG-GFP (left panel). Levels of SNCG expression were normalized to those of RPLP0 internal control. Graph shows fold enrichment over SUM-CTL-GFP cells. Data represent mean values of two independent experiments performed in triplicate. Representative immunoblot analysis (n=3) was performed on whole cell lysate for SNCG-GFP fusion protein expression using anti β-actin antibodies as a loading control (right panel). **(C)** SUM-SNCG-GFP cells showed significant decreased apoptosis after radiation treatment (12 Gy) compared to SUM-CTL-GFP cells. Flow cytometry was done to measure apoptotic and necrotic cells. Histogram shows the percentage of apoptotic cells 72 hours after irradiation (left panel). Data represent mean values ± standard error of the mean of four independent experiments. Representative experiment of flow cytometry analysis (n=4) shows the percentage of Annexin-V and propidium iodide staining of SUM-CTL-GFP and SUM-SNCG-GFP cells irradiated or not at a dose of 12 Gy (right panel). **(D)** SUM-SNCG-GFP cells showed significant increased clonogenic potential after irradiation at 4 Gy compared to SUM-CTL-GFP cells. Clonogenic cell survival assay was performed; curves and bar graph show the percentage of survival after irradiation (left panel). Representative pictures of 2-week-old colonies after fixation and crystal violet staining (right panel). Data represent mean values ± standard error of the mean of three independent experiments. ^**^ = *P value* < 0.01; ^*^ = *P value* < 0.05 ; ns = not significant.

To further evaluate the role of SNCG in the biological response of breast cancer cells to ionizing radiation, we analyzed the impact of SNCG ectopic expression in the triple-negative SUM159PT cells that do not express endogenous SNCG (Figure 1). SNCG was introduced by transfection as a fusion with the GFP protein and transfected cells were successively sorted three times by FACS on GFP expression to create a stable SUM-SNCG-GFP cell population (Figure 4A). Sorted positive SUM-SNCG-GFP cells expressed a high level of SNCG-GFP transcript compared to SUM-CTL-GFP cells (Figure 4B, left panel). The fusion protein was detected at the expected size of 45 kDa by western blotting using anti-SNCG antibody only in SUM-SNCG-GFP cells (Figure 4B, right panel). We first verified that SNCG-GFP fusion protein was functional in SUM159PT cells by showing a decreased sensitivity to Taxol treatment of SUM-SNCG-GFP cells compared to SUM-CTL-GFP cells (data not shown). After a 12 Gy-radiation treatment, the apoptosis of the SUM-SNCG-GFP cells was significantly decreased compared to the SUM-CTL-GFP cells (Figure 4C). Considering the clonogenic efficiency after irradiation, we observed an improved overall survival at 4 and 8 Gy in the SUM-SNCG-GFP cells compared to control SUM-CTL-GFP cells (Figure 4D). Altogether, these results demonstrated that ectopic expression of SNCG rendered SUM159PT cells more resistant to radiation.

### Expression of SNCG attenuates p53 signaling and increases p21^Waf/Cip1^ expression in SUM159PT cells

**Figure 5:**
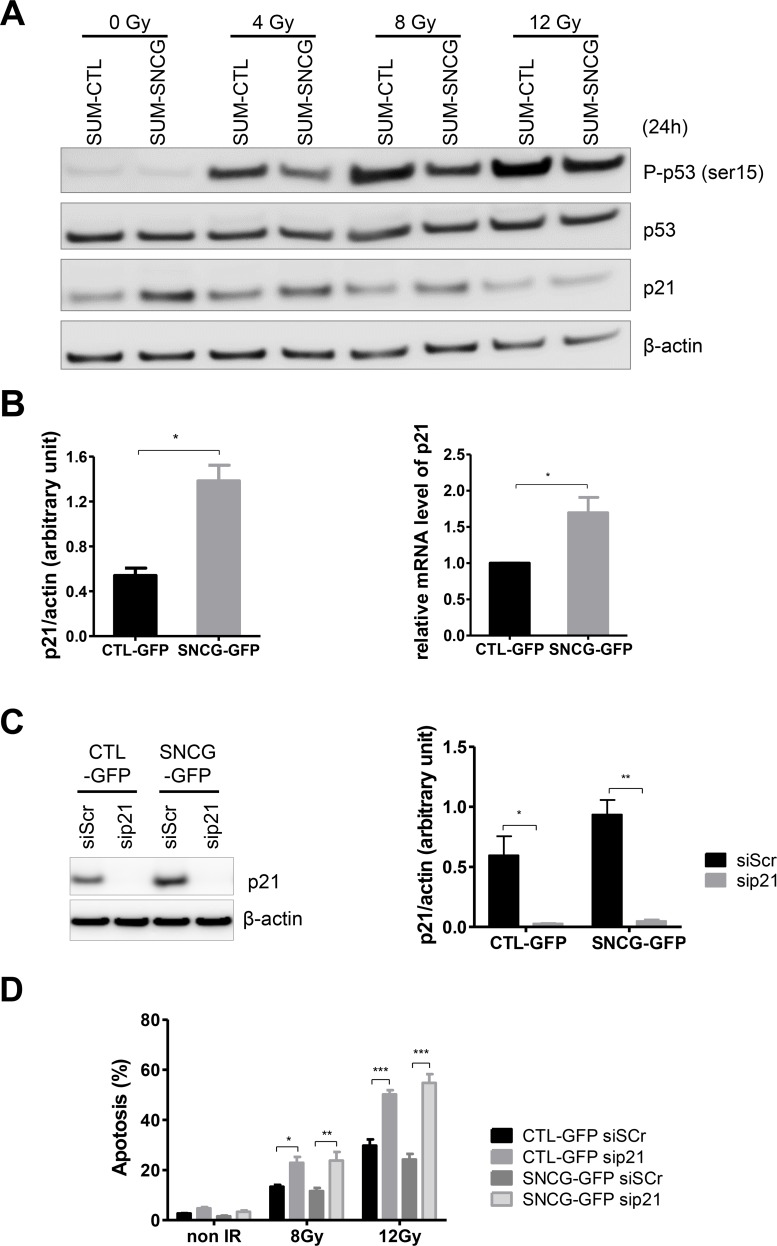
SNCG expression attenuates p53 pathway activation and increases p21^Waf1/Cip1^ expression in SUM159PT cells **(A)** Representatives pictures (n=3) of immunoblotting of phospho-p53 (Ser15), total-p53, p21, and β-actin on whole cell lysates of SUM-CTL-GFP and SUM-SNCG-GFP cells irradiated or not at a dose of 4, 8, or 12 Gy. Cells were lysed 24 hours after irradiation. **(B)** Bar graph shows quantitative analysis of scanning densitometric values of p21 protein as ratio to β-actin protein (left panel). Bar graph shows qRT-PCR analysis of p21 RNA (normalized against RPLP0) as fold enrichment over SUM-CTL-GFP cells (right panel). Data represent mean values ± standard error of the mean of three independent experiments. **(C)** sip21-treated cells showed reduced protein expression compared to siScramble (siScr)-treated cells. Representative immunoblot analysis was performed on whole cell lysate for p21 expression using anti β-actin antibody as a loading control (left panel). Bar graph shows quantitative analysis of scanning densitometric values of p21 protein as ratio to β-actin protein (right panel). Data represent mean values ± standard error of the mean of three independent experiments. **(D)** sip21-treated cells showed increased apoptosis after radiation treatment (8 and 12 Gy) compared to siScramble (siScr)-treated cells. Flow cytometry analysis was done to detect apoptotic and necrotic cells. Bar graph shows the percentage of apoptotic cells 96 hours after irradiation. Data represent mean values ± standard error of the mean of three independent experiments. ^***^ = *P value* < 0.005, ^**^ = *P value* < 0.01, ^*^ = *P value* < 0.05.

Genotoxic stress like radiation triggers a series of post-translational modifications on p53 that contribute to its stabilization, nuclear accumulation and biochemical activation. We then compared the phosphorylation status of serine 15 of activated p53 in irradiated SUM-SNCG-GFP and SUM-CTL-GFP cells. Radiation treatments induced phosphorylation of p53 to a lesser extent in SUM-SNCG-GFP cells compared to SUM-CTL-GFP cells (Figure 5A). No significant difference was observed for the expression of total p53 (Figure 5A). We then examined the expression of p21^Waf1/Cip1^, a p53-inducible protein that plays an important role in cell cycle, DNA repair, and apoptosis. Radiation did not induce a significant variation of p21 expression except at 12 Gy (Figure 5A). However, we observed an increase of p21 expression in SUM-SNCG-GFP cells as compared to SUM-CTL-GFP cells even in the absence of irradiation (Figure 5A). We confirmed that SUM-SNCG-GFP cells expressed a significantly higher level of p21 at both the protein and the RNA levels (Figure 5B). To determine whether p21 acts as a potential positive mediator of the radioprotective effect of SNCG, we evaluated the effect of p21 down-regulation on radiation sensitivity of both SUM-SNCG-GFP and SUM-CTL-GFP cells. We first confirmed that p21 siRNA effectively knocked down its protein level as determined by Western blotting in both cell populations (Figure 5C). We then evaluated the radiation sensitivity of siScr- or sip21-transfected cells. Loss of p21 significantly increased radiation-induced apoptosis at 8 and 12 Gy (Figure 5D). This dramatic effect of p21 inhibition on radiosensitivity also led to the loss of the protective effect of SNCG expression in SUM-SNCG-GFP cells as compared to SUM-CTL-GFP cells (Figure 5D). Altogether, these results suggested that in SNCG-expressing cells, activation of p53 pathway was less sustained which might render these cells less susceptible to apoptosis compared to SUM-CTL-GFP cells. On the other hand, our results demonstrated the role of p21 in radiation sensitivity in our model, which might explain some of the effects of SNCG expression on radioresistance of SUM159PT cells.

## DISCUSSION

Synuclein family members (α, β and γ) have been extensively studied since their discovery thirty years ago due to their involvement in human pathology, mainly Parkinson's disease and cancer [26, 27]. Thus, synuclein-γ (SNCG) abnormal expression has been described in a wide range of human cancer such as endometrial [28], bladder [29], prostate [30], ovarian [31], gastric [32], liver [33], lung [34], colon [35], and breast [5, 6]. In ovarian and breast carcinoma, hypomethylation of SNCG gene CpG island is responsible for aberrant SNCG expression. Tissue-specific methylation patterns were observed between breast and ovarian cancer cells [36].

SNCG has been described as playing a role in regulating resistance to chemotherapeutic agents in breast cancer, but whether SNCG modulates radiosensitivity of breast cancer cells is not known. It has been reported that SNCG expression was upregulated in irradiated human breast cancer cells and this tumor cell-secreted SNCG may contribute to immune suppressive effects [37]. However, whether this radiation-induced expression of SNCG might be implicated in the protection of the tumor cells themselves remains to be determined. Recently, expression of the RNA-binding protein HuR has been shown to upregulate SNCG expression in hepatocellular carcinoma [33]. Meanwhile, HuR expression has been shown to be associated with radioresistance of triple-negative breast cancer cells [38]. Taken together, these studies suggest a potential link between HuR/SNCG interaction and radioresistance of breast cancer cell. Here we have investigated whether modulating the level of SNCG expression could affect the radiosensitivity of breast cancer cells. We first assessed SNCG expression in various breast cancer cells and found that SNCG mRNA as well as protein were highly expressed in two luminal cell lines, T47D and MCF7. Interestingly, SNCG protein expression was also observed in ZR-75-1 cells [39]. SNCG expression in T47D cells has been described by several groups and T47D is a prominent cellular model for SNCG studies in breast cancer [7, 40, 41]. Considering SNCG expression in MCF7 cells, both none expression [15, 42] or expression [7, 43, 44] have been documented by different groups. We further tested the effects of silencing SNCG on radiosensitization of these two cell lines and observed that both T47D and MCF7 cells were radiosensitized upon down-regulation of endogenous SNCG expression. Therefore, we evaluated the effect of ectopic SNCG expression in a triple-negative breast cancer (TNBC) cell line, SUM159PT [18]. TNBC do not express estrogen receptor (ER), progesterone receptor (PR) and do not have HER-2/Neu amplification; given the lack of validated molecular targets and the poor outcome in patients with TNBC, there is a clear need for a greater understanding of TNBC [45]. We have previously used SUM159PT cells for a study of ionizing radiation effects on TNBC physiology [46]. Here we have ectopically expressed SNCG in SUM159PT and we demonstrated that SNCG expression increased their resistance to radiation. Altogether, our experiment of gain- or loss-of-function strongly suggested that SNCG is able to modulate the response of breast cancer cells to ionizing radiations.

Interestingly, Min and collaborators have recently shown that overexpression of SNCG predicts lack of benefit from radiotherapy for breast cancer patients [17]. Thus, our results are in complete agreement with this study and reinforce the idea that SNCG expression may serve as a potential biomarker to identify breast cancer patients who are less likely to benefit from radiotherapy. The relationship between SNCG expression and radiotherapy stratified survival was described in two other types of cancer, glioblastoma and lung cancer [17]. SNCG expression has been reported in several other human cancer [28, 30–32], including hepatocellular carcinoma (HCC) [33]. Since new radiotherapy techniques have expanded the indication of radiotherapy for the treatment of HCC [47], it would be of interest to determine the potential effect of SNCG in these cells.

SNCG is classified as an unstructured protein and has several potential binding partners. It is hypothesized that SNCG affects the mRNA levels of several regulatory proteins but in ways that are difficult to predict [48]. Thus, SNCG binds and mediates AP-1 activity [49, 50], activates Jun Kinase 1 (JNK1) [12], activates Estrogen Receptor (ER-α) transcription [22], interacts with androgen receptor (AR) [30], with HER2 [42], with AKT [34], with phospholipase Cβ2 [48], with PolyC binding protein 1 (PCBP1) [51]. The molecular mechanisms of how SNCG favors radioresistance remain to be determined and further research is required to unveil those mechanisms. Importantly, it has been proposed that the role of synuclein family proteins may change in response to stress or changing environmental conditions, like regulating gene expression [27]. In our study, we showed that enforced SNCG expression was able to activate p21^Waf1/Cip1^ transcription and to increase p21 protein expression in SUM159PT cells. Several reports have shown that p21 has other functions than cyclin-dependent kinases inhibition, especially in the DNA damage response [52]. It could play an important role in keeping cells alive after DNA damage and p53 pathway activation, in order to ensure correct DNA repair [53]. Cells lacking p21 or treated with p21 antisense display enhanced sensitivity toward apoptosis induced by DNA-damaging agents [54, 55], whereas tumors expressing high levels of p21 are prone to be resistant to DNA-damaging agents [56, 57]. Thus, p21 could limit the effectiveness of chemo- and radio-therapies and it appears then as a therapeutic target in breast and other cancers [58]. This was supported by our data showing that down-regulation of p21 markedly increased radiosensitivity of SUM159PT breast cancer cells. This suggests that p21 could be implicated in SNCG pathway in some breast cancer cells and could play a role in chemo- and radioresistance induced by SNCG expression. Further studies are warranted to confirm this model.

In conclusion, our study demonstrated for the first time that SNCG confers radioresistance to breast cancer cells. Our data support its potential as a biomarker that predicts the effectiveness of radiotherapy. Moreover, we shed light on the possible interaction between SNCG and p21, which may contribute to the radioresistance of breast cancer cells. Given the importance of radiation therapy to the treatment of early-stage breast cancer and other cancers, further evaluations of SNCG effects and its mechanisms on radiation sensitivity will be important.

## MATERIALS AND METHODS

### Cell culture

All cell lines used in this study were originally obtained from the American Type Culture Collection. MCF7 cells were cultured in Dulbecco's modified Eagle's medium (Gibco) supplemented with 10% of fetal bovine serum (FBS, Gibco) and Zell Shield (Minerva Biolabs, Biovalley). T47D cells were cultured in RPMI 1640 medium (Gibco) supplemented with 10% FBS and Zell Shield. SUM159PT (hereby named SUM) cells were cultured in Ham's F12 Nutrient Mixture (Gibco) supplemented with 5% FBS, hydrocortisone (1μg/ml) (Sigma-Aldrich), insulin (5μg/ml) (Sigma-Aldrich) and Zell Shield. All cell cultures were maintained at 37°C in a humidified incubator in an atmosphere of 5% CO_2_ – 95% air.

### Plasmids and siRNA transfections

To establish stable cell lines expressing SNCG, SUM cells were transfected with a pCMV6-SNCG-GFP plasmid DNA (Origene, RG204173) or with the corresponding empty vector pCMV6-A-GFP plasmid DNA (Origene, PS100010) as a control. Briefly, one day before transfection, 2×10^5^ SUM cells/well were plated in a 6-well plate. Cells were transfected with 1.5 μg/well of the recombinant plasmid or the empty plasmid as control, respectively, using jetPRIME^®^ (Polyplus) according to the manufacturer's transfection protocol. Fresh growth medium was replaced after 24 h of transfection. Between 5 and 7 days after transfection, GFP-positive SUM cells were successively sorted three times by using a FACSAria II cell sorter (BD Biosciences) to a purity of >85%, and analyzed.

For siRNA experiments, T47D and MCF7 cells were transfected with SNCG siRNA and control siScramble (siSrc) constructs (Origene, SR304497) and SUM-SNCG-GFP and SUM-CTL-GFP cells were transfected with p21 siRNA and control siScr constructs (Origene SR300740), using jetPRIME^®^ (Polyplus) according to the manufacturer's transfection protocol. Total RNA and proteins were extracted 48 h after transfection for analysis. For irradiation experiments, cells were irradiated 48 hours after transfection.

### RNA isolation and quantitative RT-PCR

RNA extraction and quantitive PCR were performed as previously described [59]. Briefly, total RNA was isolated using the RNeasy Plus Extraction kit (Qiagen). Reverse transcription (RT) was performed using QuantiTect Reverse Transcription kit (Qiagen) on 1 μg of RNA, according to the manufacturer's protocol. Quantitative RT-PCR (qRT-PCR) was achieved on Stratagene Mx3005P (Agilent Technologies) using KAPA SYBR^®^ Fast Universal qPCR kit (Kapa Biosystems). The thermal cycling program was 95°C for 20 sec, 60°C for 30 sec and 72°C for 30 sec during 40 cycles. Experiments were performed in duplicate, and the comparative threshold cycle method was used for the calculation of amplification fold (2−ΔΔCT method). Primer sequences are presented in Table 1.

**Table 1:** oligonucleotide primer sequences used in qPCR

Gene	Primer sequence
RPLP0 Forward RPLP0 Reverse	GTGATGTGCAGCTGATCAAGACT GATGACCAGCCCAAAGGAGA
SNCG Forward SNCG Reverse	GGTCATGTATGTGGGAGCC CACTTCCTCTTTCTCTTTGG
P21 Forward P21 Reverse	ACCATGTGGACCTGTCACTGT TTAGGGCTTCCTCTTGGAGAA

### Western blot analysis and antibodies

Cells were harvested and lysed on ice with a lysis buffer (20 mM HEPES pH7.4, 142 mM KCl, 5 mM MgCl_2_, 1 mM EDTA, 5% glycerol, 1% NP40 and 0.1% SDS) supplemented with freshly added protease and phosphatase inhibitors (#P8340 and #P5726, respectively, Sigma). Cells then were disrupted by centrifugation 10 min at 10,000 g at 4°C. The protein concentrations of the supernatant were quantified by bicinchoninic acid (BCA) protein assay kit (Pierce^®^, ThermoFisher). Total proteins (30-40 μg) were separated on a NuPAGE™ 4-12% Bis-Tris Gel (ThermoFisher) and electrophoretically transferred onto PolyVinylidene fluoride (PVDF) membranes (Merck Millipore). Membranes were blocked at room temperature in a blocking buffer (phosphate-buffered saline (PBS) containing 0.2% of casein (VWR E666) and 0.05% Tween 20) and probed with indicated primary antibodies overnight at 4°C. Membranes were washed with PBS-0.05% Tween 20 for 30 min and incubated with secondary antibodies conjugated with HorseRadish Peroxydase (HRP) for 1 hour. Protein-antibody complexes were visualized by chemoluminescence with the SuperSignal^®^ West Dura Extended Duration Substrate (Thermo scientific), using a LAS-3000 imaging system (Fujifilm, Tokyo, Japan) or X-ray films (CL-Xposure TM Film, Thermo scientific). Primary antibodies were SNCG (Abcam, ab55424, 1:1000), β-actin (Santa-Cruz, sc-47778, 1:200), phosphor-p53 (Ser15) (Cell Signaling, #9284, 1:1000), p53 (Cell Signaling, #2524, 1:1000), p21 Waf1/Cip1 (Cell Signaling, #2947, 1:1000), Bax (Santa-Cruz, sc-7480, 1:1000) and Bcl-xL (Cell Signaling, #2764, 1:1000).

### Immunofluorescence staining

Cells were cytocentrifuged on glass slides, air-dried, washed with PBS and fixed in 4% paraformaldehyde (PFA) for 15 min at room temperature (RT). After washing with PBS, cells were permeabilized with 0.5% Triton X100 in PBS, washed with PBS and non-specific binding was blocked by 5% FBS in PBS for 45 min at RT. Cells were incubated with or without SNCG antibodies (Santa-Cruz, sc135676, 1:100) for 45 min at RT in blocking buffer. Cells were washed and incubated with secondary antibody Alexa Fluor 594 F(ab’)2 fragment of goat anti-rabbit (Life Technologies A11072, 1:1000), counterstained with DAPI nuclear stain and mounted in Prolong Gold (Invitrogen). Optical sectioning images were taken using AxioImager Z1-Apotome (Zeiss, Germany). ZEN software (Zeiss) was used for microscope image analysis.

### Cell irradiation treatment

Cells were seeded in 6-well plates at a density of 10^5^/well and grown overnight and irradiated with a dose rate of 1 Gy/min, with graded doses (4-12 Gy). A Darpac 2000 X-ray unit (Gulmay Medical Ltd, Shepperton, UK), operated at 80 kV, 8 mA, using 2.3 mm Al filtration was used for irradiation.

### Apoptosis detection

APC Annexin V Apoptosis Detection Kit with PI (Biolegend) was used as a method to measure radiation-induced cytotoxicity. The assay was performed according to the manufacturer's instructions. Briefly, 72h after irradiation, cells were harvested and washed twice with cold Cell Staining Buffer (Biolegend), resuspended in Annexin V and propidium iodide (PI) staining solution and then analyzed immediately by flow cytometry (BD FACS Canto II).

### Clonogenic assay

For irradiation experiments, cells were seeded in 6-well plates at a density of 10^5^ cells per well and grown overnight. After irradiation, cells were harvested the next day using trypsinization, counted and a specific number of cells (100, 500 and 1000 cells) was plated in 6-well plates in triplicate for clonogenic assay. After 10 to 12 days, the medium was removed and cells were rinsed with PBS. Colonies were fixed with 4% PFA solution for 10 min at room temperature and were stained with 1% crystal violet solution in water. Plates were rinsed with water and left for drying at room temperature. Colonies (≥50 cells) were counted on the following day and survival fraction was calculated as the ratio of the plating efficiency of the treated cells to that of control cells.

### Cell cycle analysis

Cells were harvested with trypsin, washed with PBS and fixed in 70% ethanol at -20°C. Fixed cells were stained with buffer including 20 μg/ml PI (Sigma-Aldrich) and 50μg/ml RNase A (ThermoFisher) for 20 min at 37°C in the dark. DNA content was analyzed by measuring the intensity of the fluorescence produced by PI using FACSCanto II flow cytometer (BD Biosciences) and analyzed by FlowJo (Tree Star). The cell-cycle distribution was evaluated by counting >10,000 cells for each sample.

### SA-β-Galactosidase assay

Senescent cells were identified by using Senescence Cells Histochemical Staining Kit (Sigma-Aldrich) according to the manufacturer's protocol. Briefly, 72h after irradiation, MCF7 cells were fixed using fixation buffer for 7 min and incubated with X-Gal-containing reaction mixture. Incubation time was 12-14 h and SA-β-Gal-positive cells were counted in >8 independent microscopic fields for a total of at least 500 cells for each case in all experiments.

### Multiple resazurin assay

Irradiated and control cells were harvested 24h after irradiation using trypsinization, counted and 2500 cells were plated in 96-well-plates in six replicates. For multiple resazurin assay, the culture medium was replaced by 200 μl Hank's Balanced Salt Solution (HBSS, Gibco) containing 10% v/v resazurin (7-Hydroxy-3H- phenoxazin-3-one 10-oxide, Sigma-Aldrich) and fluorescence was measured (excitation 530nm, emission 590nm) by using EnVision Multilabel Plate Reader (PerkinElmer). The proliferation survival was calculated as previously described [23].

### Statistical analysis

Data are expressed as mean values ± standard deviation of at least 3 independent experiments. The statistical analysis was done by using Student's *t*-test or two-way ANOVA test and the p value < 0.05 was considered significant.

## SUPPLEMENTARY MATERIALS FIGURE


