# Supplementary material for: Synuclein gamma expression enhances radiation resistance of breast cancer cells
Source: Oncotarget. 2018 Jun 8;9(44):27435–47. doi: 10.18632/oncotarget.25415 (PMC6007952; doi:10.18632/oncotarget.25415)
Supplement: Supplementary file 1 [file oncotarget-09-27435-s001.pdf]

Synuclein gamma expression enhances radiation resistance of breast cancer cells

SUPPLEMENTARY MATERIALS

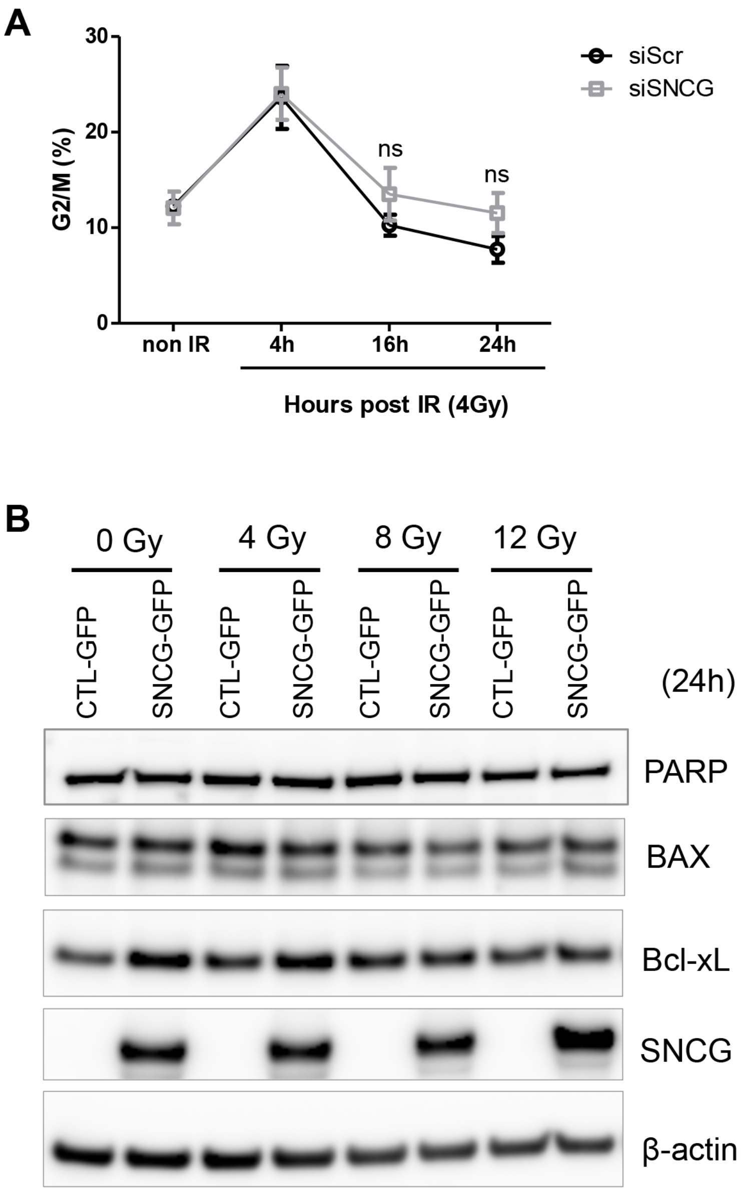

Supplementary Figure 1: (A) Analysis of the effect of radiation (IR) on MCF7 cell cycle by flow cytometry. siSNCG- or siScrtreated MCF7 cells were exposed to 4 Gy of radiation, incubated for the indicated hours, and analyzed for cell cycle by flow cytometry. Results depict the percentage of cells in G2/M phase of the cell cycle and represent the mean ± SD of three independent experiments. ns = not significant. **(B)** Analysis of the effect of radiation on apoptosis signaling genes expression. Representative immunoblot analysis (n=3) of apoptosis signaling genes expression after radiation. SUM-CTL-GFP and SUM-SNCG-GFP cells were exposed to radiation at the indicated doses, incubated for 24 hours, and analyzed for levels of full-length PARP, BAX, Bcl-xL and SNCG by immunoblotting. β-actin expression was used a a loading control.
